# Therapeutic Effect of Human Umbilical Cord Mesenchymal Stem Cells at Various Passages on Acute Liver Failure in Rats

**DOI:** 10.1155/2018/7159465

**Published:** 2018-11-14

**Authors:** Yongting Zhang, Yuwen Li, Wenting Li, Jie Cai, Ming Yue, Longfeng Jiang, Ruirui Xu, Lili Zhang, Jun Li, Chuanlong Zhu

**Affiliations:** ^1^Department of Infectious Disease, The First Affiliated Hospital of Nanjing Medical University, Nanjing 210029, China; ^2^Department of Pediatrics, The Affiliated Hospital of Yangzhou University, Yangzhou 225000, China; ^3^Department of Pediatrics, The First Affiliated Hospital of Nanjing Medical University, Nanjing 210029, China; ^4^Third Liver Unit, Department of Infectious Disease, The First Affiliated Hospital of Science and Technology of China, Hefei 230001, China

## Abstract

Recent studies have described beneficial effects of an infusion of mesenchymal stem cells (MSCs) derived from Wharton's jelly tissue, for the treatment of acute liver failure (ALF). However, data on the therapeutic potential of culture-expanded MSCs are lacking. We examined the therapeutic potential of passage five (P5) and ten (P10) human umbilical cord- (hUC-) MSCs via their transplantation into Sprague-Dawley (SD) rats with D-galactosamine (D-GalN) and LPS-induced acute liver failure (ALF). SD rats were randomly divided into three groups: control group, P5 hUC-MSCs group, and P10 hUC-MSCs group. After transplantation, P5 hUC-MSCs provided a significant survival benefit. The analysis of aspartate aminotransferase (AST), alanine aminotransferase (ALT), and total bilirubin (TBIL) levels showed that transplantation with P5 hUC-MSCs was more effective than treatment with P10 hUC-MSCs. P5 hUC-MSCs also successfully downregulated the hepatic activity index (HAI) scores. Compared to P10 hUC-MSCs *in vivo*, P5 hUC-MSCs significantly enhanced the regeneration and inhibited the apoptosis of hepatocytes. CM-Dil-labeled hUC-MSCs were found to engraft within the recipient liver, whereas the homing of cells to the recipient liver in the P10 hUC-MSCs group was less effective compared to the P5 hUC-MSCs group. Previous studies have shown that the concentration of hepatocyte growth factor (HGF) in the injured liver was significantly increased. HGF is commonly known as the ligand of c-Met. The level of c-Met in hUC-MSCs as detected by Western blotting indicated that at a higher passage number, there is a decrease in c-Met. These data suggest that direct transplantation of P5 hUC-MSCs can more efficiently home to an injured liver. Subsequently, the P5 hUC-MSCs can rescue ALF and repopulate the livers of rats through the stimulation of endogenous liver regeneration and inhibition of hepatocellular apoptosis for compensated liver function, which is dependent on the higher level of c-Met than P10 hUC-MSCs.

## 1. Introduction

Liver failure is a clinical syndrome characterized by jaundice, ascites, hepatic encephalopathy, and a bleeding tendency due to the impaired liver function. The syndrome can be caused by a variety of factors such as viral hepatitis, autoimmune hepatitis, drug-induced liver injuries, metabolic diseases, and circulatory disturbances [1].

To date, the only therapeutic option for liver failure is orthotopic liver transplantation. Reasons for failure to receive transplantation have included lack of available organs, high expense, and the requirement for lifelong immunosuppressive medication. Other treatment strategies include bioartificial livers that are short of liver cells and medical management [2]. Consequently, there is an urgent need for novel therapeutic options. Saunders et al. suggested that liver failure results from the catastrophic consequences of liver loss and the regeneration of remnant hepatocytes fails to repair in a timely manner, resulting in higher mortality rates [3]. Cell-based therapy in regenerative medicine shows considerable promise. Mesenchymal stem cells (MSCs) have a number of advantages, including their self-renewal, proliferation, and differentiation properties. MSCs were originally found in bone marrow and can also be isolated from the umbilical cord, adipose tissue, dental pulp, and amniotic fluid [4, 5]. MSCs, which are easily acquired and devoid of any ethical contentions, are preferred for autografts, especially cells from the bone marrow (BM). The moderate efficacy of autologous BM-MSCs is thought to be due to their aging and deficiency in vitality [6].

Recently, several studies reported that human umbilical cord mesenchymal stem cells (hUC-MSCs) are another promising source, as they are free from aging and are unlimited in their supply. They are also characterized by lower immunogenic potency, higher proliferative activity, and multipotency [7]. Gholamrezanezhad et al. [8] have shown that there was no significant improvement in liver function after a 1-month period of follow-up because the homing ability of BM-MSCs into the liver occurred in only a limited number of infused cells. Peng et al. also reported that the homing ability of MSCs is the main reason why autologous MSC transplantation did not achieve acceptable long-term effects on the prognosis of a patient [9]. The lingering problem pertaining to cell-based therapies is whether the delivered cells home to the injured sites and how to improve their homing ability.

Hepatocyte growth factor (HGF), which is the most effective mitogen for hepatocytes, functions via a receptor termed the cellular mesenchymal to epithelial transition factor (c-Met) [10]. The HGF-c-Met axis has been extensively examined, as it is crucial for MSC homing during liver injury [11]. Our primary experiment found that levels of HGF in the liver significantly increased and peaked at 24 h and 48 h, respectively, post-D-GalN/LPS injection. Meanwhile, overexpressed c-Met in BM-MSCs improved the homing efficacy to the injured liver and improved the liver function [12]. Furthermore, freshly isolated MSCs lose ligands or receptors, which respond to migratory signals during expansion [13].

In this study, we aimed to investigate the effect of CM-Dil-labeled hUC-MSCs on liver failure and the underlying mechanism of homing among various passages in hUC-MSCs.

## 2. Materials and Methods

### 2.1. Animals

Male Sprague-Dawley rats weighing 220 to 250 g were purchased from the Animal Laboratory Center of Nanjing Medical University (Nanjing, China). All procedures were performed in strict accordance with the recommendations in the Guide for the Care and Use of Laboratory Animals of Nanjing Medical University. All experimental protocols were approved by the Animal Ethical and Welfare Committee of Nanjing Medical University (Nanjing, China).

### 2.2. hUC-MSCs

P2 hUC-MSCs were purchased from Cyagen. The cell pellet was cultured in an expansion medium composed of the human umbilical cord mesenchymal stem cell basal medium (HUXUC-01001, Cyagen, China), supplemented with 10% human umbilical cord mesenchymal stem cell cell-qualified fetal bovine serum, 1% glutamine, and 1% penicillin-streptomycin (Cyagen), at 37°C, with 5% carbon dioxide in a fully humidified environment.

Native hUC-MSCs (at P5) were suspended at a concentration of 1 × 10^6^ cells/mL, washed twice in phosphate-buffered saline (PBS), and then incubated for 30 min at 4°C in the dark with the following anti-human antibodies conjugated with fluorescein isothiocyanate (FITC) or phycoerythrin (PE): anti-CD31-PE (303105), anti-CD45-PE (368509), anti-CD34-PE (343605), anti-CD44-FITC (338803), anti-CD90-FITC (328107), and anti-CD105-FITC (328107; all from BioLegend). After 30 min, cells were washed and resuspended in 300 mL Cell Fix (BD). PE-conjugated IgG1 and FITC-conjugated IgG1 were used as isotype controls (R&D Systems Inc. and Santa Cruz Biotechnology Inc.). Immunophenotyping of hUC-MSCs was performed by flow cytometry.

### 2.3. Animal Model and Cell Transplantation

The animal model used was described in a previous study [14]. D-galactosamine (D-GalN, 1000 mg/kg body weight, Sigma) and lipopolysaccharide (LPS, 10 *μ*g/kg body weight; Sigma) were dissolved in PBS and simultaneously administered to animals intraperitoneally. Cells were transplanted under 10% chloral hydrate injection anesthesia, 24 h after the administration of D-GalN/LPS. SD rats were randomly divided into three groups: group A, rats that received 1 mL of normal saline via tail vein as the control (*n* = 12); group B, rats that received 1 mL of P5 hUC-MSCs (1 × 10^7^ cells/kg) via tail vein (*n* = 12); and group C, rats that received P10 hUC-MSCs (1 × 10^7^ cells/kg) via tail vein (*n* = 12). Blood samples were collected at 24 h, 48 h, and 72 h after the injection of D-GalN/LPS, and finally, entire livers were collected, fixed, then prepared for further analysis.

### 2.4. Serum Transaminase Levels

Serum samples were stored at −80°C until the time of analysis. Serum aspartate aminotransferase (AST), total bilirubin (TBil), and alanine aminotransferase (ALT) levels in the rat blood were measured according to the manufacturer's directions with a kit from the Nanjing Jiancheng Bioengineering Institute (Nanjing, China).

### 2.5. Liver Histology

Formalin-fixed, paraffin-embedded liver samples were sectioned at a thickness of 4 *μ*m and stained with hematoxylin & eosin (H&E). Histological assessment was performed using the hepatic activity index (HAI) and was graded following guidelines previously described [15].

### 2.6. Cell Labeling

Before labeling, the P5 and P10 hUC-MSCs in plates were trypsinized for cell counting. CM-Dil (Invitrogen, USA) was then directly added to the culture medium to label cells in the working solution (2 *μ*g/mL) for 5 minutes at 37°C, and then for an additional 15 minutes at 4°C. Following labeling, the medium was discarded, and the cells were washed three times with PBS to eliminate residual fluorescent-conjugated antibodies. At 24 h after the injection of D-GalN/LPS, the labeled P5 and P10 hUC-MSCs (1 × 10^6^ cells in 1 mL of normal saline) were transplanted into the rats, and the control rats received 1 mL of normal saline. After an additional 24 h, the rats were killed so the livers could be removed. The frozen sections were examined by fluorescence microscope and analyzed by Image-Pro Plus.

### 2.7. Immunohistochemistry

The sections of formalin-fixed tissue were left for 1 hour at 60°C before deparaffinized, rehydrated, and blocked in 3% hydrogen peroxide in ethanol for 15 minutes. For proliferating cell nuclear antigen (PCNA) immunohistochemistry, sections were blocked with 1.5% bovine serum albumin (BSA) for 30 minutes and incubated with anti-mouse PCNA (Google biology, Wuhan) overnight at 4°C. Sections were washed and incubated with a rabbit anti-human IgG secondary antibody (K5007, DAKO, Denmark) for 50 minutes at room temperature. The sections were then added to a newly prepared DAB kit (DAKO, Denmark). The positive nucleus is indicated with brown and yellow colors. Sections were counterstained with hematoxylin for 3 minutes and 1% of hydrochloric acid for several seconds. Finally, the sections were observed under a Nikon light microscope after being dehydrated. For terminal deoxynucleotidyl transferase-mediated nick-end labeling (TUNEL) staining, we used the In Situ Cell Death Detection Kit (Roche, Switzerland) according to the vendor's instructions.

### 2.8. Western Blot Analysis of c-Met Protein

To detect the level of c-Met, the total protein samples in the same quantity for P3, P5, P8, and P10 hUC-MSCs were extracted. Proteins were identified based on instructions provided with the BCA Kit (Thermo Scientific, USA) and then separated by sodium dodecyl sulfate-polyacrylamide gel electrophoresis (SDS-PAGE) and transferred onto nitrocellulose membranes. The membranes were incubated with mouse monoclonal anti-c-Met antibody (Santa Cruz Biotechnology, Santa Cruz, USA) at a 1 : 500 dilution overnight at 4°C and then incubated with a sheep anti-mouse immunoglobulin G secondary antibody (GE Healthcare Biosciences, USA) for 60 minutes at room temperature. The results were detected using an enhanced chemiluminescence (ECL) kit (GE Healthcare Biosciences, USA) and visualized under an imaging system.

### 2.9. Statistics

The data are expressed as mean ± standard deviation (SD) using SPSS version 16.0 (Chicago, IL, USA). Statistical significance was determined via a one-way analysis of the variance (ANOVA) and the log-rank test for survival analysis among the three groups. A *P* value of <0.05 was considered to be statistically significant.

## 3. Results

### 3.1. Identification of Cultured hUC-MSCs

**Figure 1:**
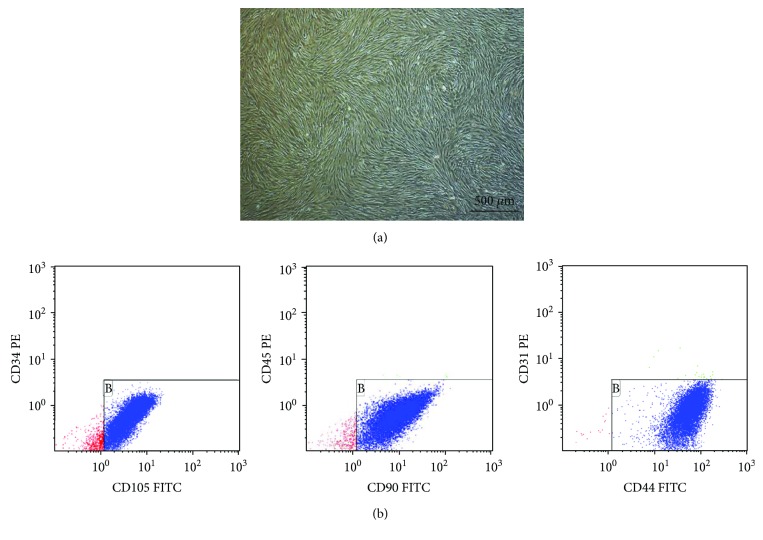
Characterization of hUC-MSCs. (a) Morphology of passage 3 hUC-MSC identification of human umbilical cord mesenchymal stem cells. (b) Flow cytometric analysis showing the immunophenotype of the passage 5hUC-MSCs. The cells were positive for CD105 (96.4%), CD44 (99.6%), and CD90 (97.6%), but negative for CD34, CD31, and CD45.

After resuscitation and cultivation for 5–7 days, cells started to present a fibroblast-like phenotype. After 14 days, the number of cells increased and grew in a parallel arrangement. After serial passage, they proliferated rapidly *in vitro*, without significant changes in morphology. Flow cytometry revealed that these cells were negative for the expression of hematopoietic markers such as CD45, CD34, and CD31. However, they were positive for CD105, CD90, and CD44, which are generally considered to be markers of MSCs. This indicated that these cells were hUC-MSCs (Figure 1).

### 3.2. Improved Survival Rate and Liver Function of ALF Rats That Received Transplanted hUC-MSCs

**Figure 2:**
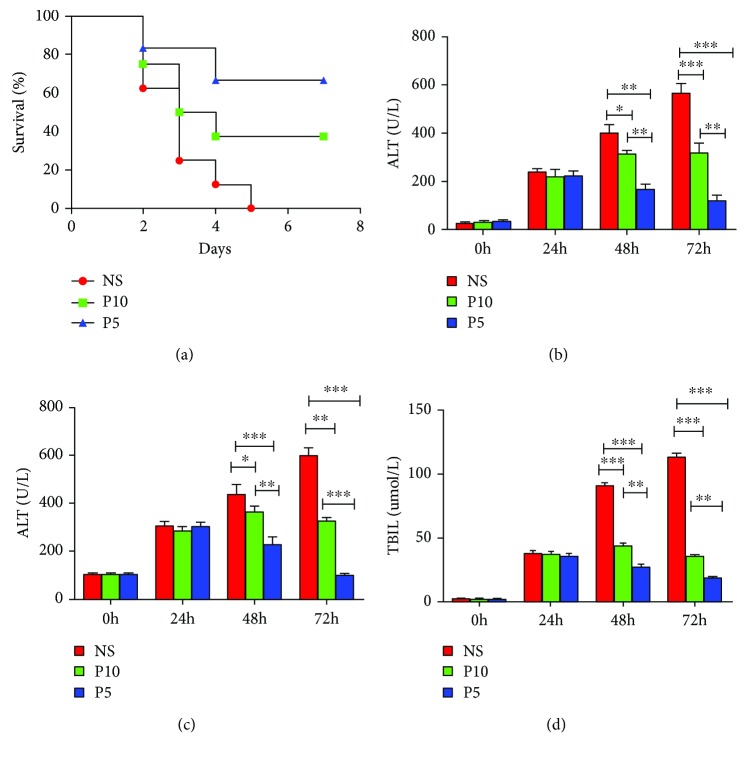
Effects of hUC-MSC transplantation on the survival rates and liver function in ALF rats. A total of 36 rats were randomly divided into three groups: P5 hUC-MSCs group, P10 hUC-MSCs group, and NS group (*n* = 12 per group). D-GalN/LPS were administered to rats intraperitoneally to induce ALF. 24 h later, the P5 hUC-MSCs and P10 hUC-MSCs (dose: 1.0 × 10^7^ cells/kg/mL) were transplanted, respectively, while the NS group was given 1 mL normal saline. The blood samples were collected at 0 h, 24 h, 48 h, and 72 h post-D-GaIN/LPS injection. (a) The survival rate in the P5 hUC-MSCs, P10 hUC-MSCs, and NS groups. Levels of serum (b) alanine transferase (ALT), (c) aspartate aminotransferase (AST), and (d) total bilirubin (TBil) in treatment of ALF rats. Data were expressed as mean ± SD. ^∗^*P* < 0.05, ^∗∗^*P* < 0.01, ^∗∗∗^*P* < 0.001. hUC-MSCs, human umbilical cord-derived mesenchymal stem cells; D-GalN, D-galactosamine; LPS, lipopolysaccharide.

All rats in the control group died between 48 h and 96 h after the saline injection. The survival rate was 37.5% in the P10 hUC-MSCs group, with three rats surviving after a week. Rats treated with the P5 hUC-MSCs had a survival rate of 62.5% at 7 days, and only three rats died. A significantly higher survival rate was observed for the P5 hUC-MSCs group (Figure 2(a)).

As shown in Figures 2(b)–2(d), the serum ALT, AST, and TBil levels in the P5 hUC-MSCs group decreased 24 h after transplantation (165.50 ± 22.73 U/L, 254.69 ± 22.89 U/L, and 27.03 ± 2.92 *μ*mol/L, respectively), while the liver function of the P10 hUC-MSCs group still increased (312.13 ± 14.98 U/L, 382.70 ± 23.48 U/L, and 35.98 ± 2.10 *μ*mol/L, respectively). 48 h after transplantation, the serum levels in the P10 hUC-MSCs group were also dramatically decreased (ALT: 269.50 ± 24.16 U/L, AST: 325.07 ± 33.38 U/L, and TBil: 35.98 ± 2.10 *μ*mol/L), with indicators of liver injury that were still lower than those of the rats that received the P5 hUC-MSCs (ALT: 118.56 ± 23.05 U/L, AST: 114.47 ± 23.06 U/L, and TBil: 18.67 ± 2.33 *μ*mol/L). Moreover, significant differences in serum levels of ALT, AST, and TBil between the P5 hUC-MSCs group and P10 hUC-MSCs group were observed at 48 h and 72 h post-D-GalN/LPS injection (*P* < 0.01). No significant differences were observed at 24 h after the D-GalN/LPS coinjection.

### 3.3. hUC-MSCs Therapy Improves Gross and Microscopic Liver Histopathology and Reduces Inflammatory Cell Infiltration

**Figure 3:**
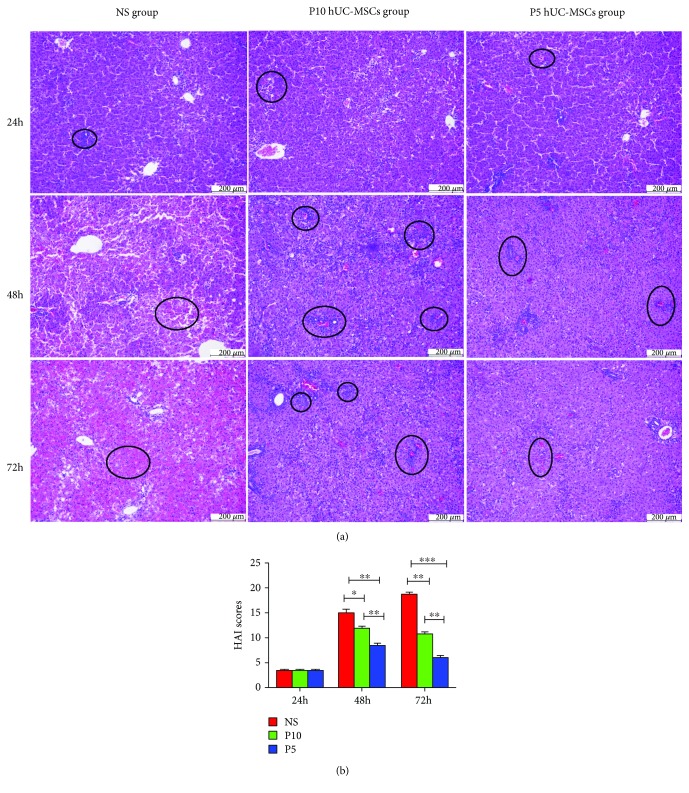
Histopathological recovery of D-GaIN/LPS-induced ALF transplanted with hUCMSCs at 24 h, 48 h, and 72 h. (a) H&E staining showed that there were massive necrosis and hepatic lobule damage in the NS group. In contrast, hepatocyte necrosis was suppressed in both P5 hUC-MSCs group and P10 hUC-MSCs group after transplantation. hUC-MSCs treatment also reduces the number of inflammatory infiltration. The black circles represent the hepatocyte necrosis and severe inflammatory cell infiltration. Bar = 200 *μ*m. (b) Histopathological grading of necrosis and inflammation of the liver sections. Data are presented as mean ± SD. ^∗^*P* < 0.05, ^∗∗^*P* < 0.01, ^∗∗∗^*P* < 0.001. D-GalN, D-galactosamine; HAI, hepatic activity index; IP, intraperitoneal; LPS, lipopolysaccharide; NS, normal saline.

To evaluate whether treatment with P5 hUC-MSCs regenerated the injured liver to a greater extent than with P10 hUC-MSCs, we further assessed the liver histology with H&E staining. H&E-stained sections revealed massive necrosis and hepatic lobule damage in the control group at 72 h, but hepatocyte necrosis was suppressed in both the P5 hUC-MSC and P10 hUC-MSC groups (Figure 3(a)). While microscopic evaluation of the H&E-stained liver sections revealed significant cytoplasmic vacuolization and severe inflammatory cell infiltration at 48 h post-P10 hUC-MSC transplantation, the sections from rats that received P5 hUC-MSC transplantation demonstrated moderate periportal inflammatory cell infiltration and hepatocyte edema at 48 h after P5 hUC-MSC transplantation (Figure 3(a)). Furthermore, HAI scores randomly assessed by expert pathologists were significantly different between necrosis and inflammatory cell infiltration (*P* < 0.05; Figure 3(b)), indicating that there was less necrosis and milder inflammatory reactions in the P5 hUC-MSC group compared to the P10 hUC-MSC group.

### 3.4. hUC-MSCs Inhibit Hepatocellular Apoptosis In Vivo

**Figure 4:**
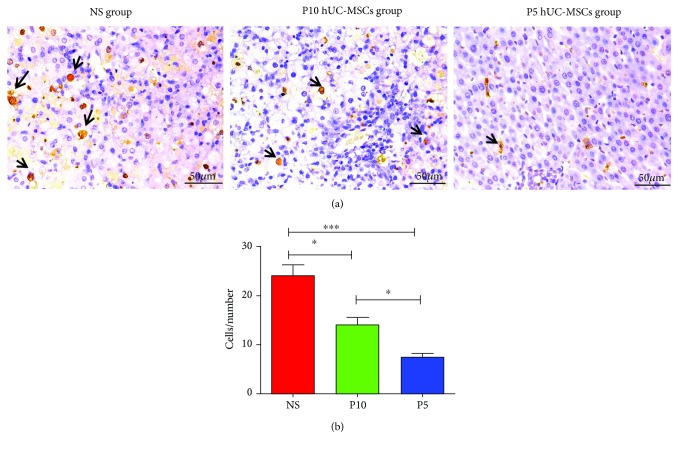
Infusion of hUC-MSCs decreases levels of apoptosis in livers D-GaIN/LPS-induced ALF. (a) Liver sections were stained by TUNEL (dark brown nuclei). Representative images from NS-treated, P10 hUC-MSC-treated, and P5 hUC-MSC-treated rats. Bar = 50 *μ*m. (b) Quantification of TUNEL-positive hepatocyte-nuclei by digital image analysis. Data are reported as mean ± SD. ^∗^*P* < 0.05, ^∗∗∗^*P* < 0.001. TUNEL, terminal deoxynucleotidyl transferase-mediated nick-end labeling.

To determine whether different passages of hUC-MSCs have an impact on hepatocellular apoptosis, the number of TUNEL-reactive hepatocyte nuclei in liver sections was determined. Four 400x fields were randomly selected from the three groups, and the number of accumulated apoptotic cells was counted using Image-Pro Plus software. Many apoptotic hepatocytes were observed in the control samples (NS: 24.0 ± 4.8), while a significant reduction was present with hUC-MSC treatment (P10: 14.0 ± 3.4; P5: 7.4 ± 1.8; Figure 4(a)). Quantification of TUNEL revealed that P5 hUC-MSC treatment effectively reduces hepatocellular death post coinjection of D-GalN and LPS when compared with P10 hUC-MSCs (Figure 4(b)).

### 3.5. hUC-MSCs Enhance Liver Regeneration

**Figure 5:**
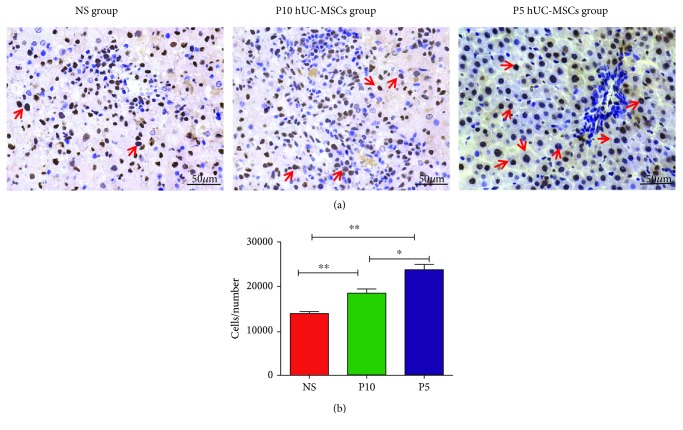
Infusion of hUC-MSCs enhances liver regeneration in livers D-GaIN/LPS-induced ALF at 48 h. (a) Liver sections were stained for PCNA (dark brown nuclei). Representative images from NS-treated, P10 hUC-MSC-treated, and P5hUC-MSC-treated rats. Bar = 50 *μ*m. (b) Quantification of PCNA-positive hepatocyte-nuclei by Image-Pro Plus software. Data are reported as mean ± SD. ^∗^*P* < 0.05, ^∗∗^*P* < 0.01, ^∗∗∗^*P* < 0.001. PCNA, proliferating cell nuclear antigen.

To evaluate whether hUC-MSCs promoted liver regeneration, the number of PCNA-positive hepatocytes was quantified and compared with control saline-treated animals. Few PCNA-positive hepatocytes were observed in the control, while many were seen after hUC-MSC administration (Figure 5(a)). In the P5 hUC-MSC group, a significant increase in the number of proliferating cells (IOD: 24604.99 ± 1132.43) was found, compared to the control (IOD: 13929.3 ± 813.7). In addition, the number of PCNA-positive cells in the P10 hUC-MSC group also increased slightly (IOD: 188550.99 ± 1654.58). These results provide evidence that infusion with MSCs enhances liver regeneration programs during ALF (Figure 5(b)).

### 3.6. CM-Dil-Labeled hUC-MSCs In Vivo

**Figure 6:**
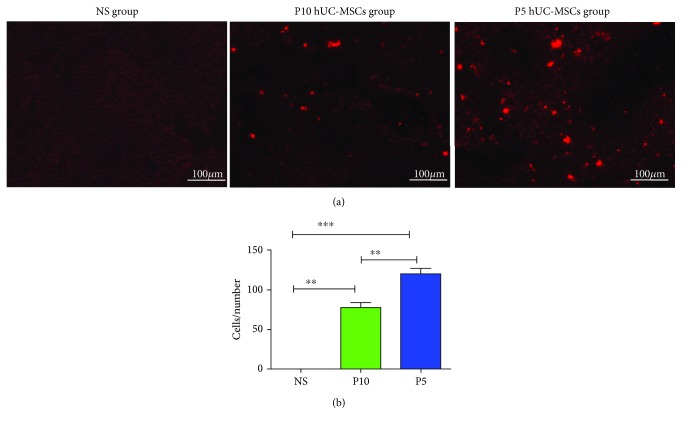
Infusion of CM-Dil-labeled hUC-MSCs enhances homing to livers D-GaIN/LPS-induced ALF at 48 h. (a) Liver sections were observed by fluorescence microscope. Representative images from NS-treated, P10 hUC-MSC-treated, and P5 hUC-MSC-treated rats. Bar = 100 *μ*m. (b) Quantification of the accumulated hUC-MSCs by Image-Pro Plus software. Data are reported as mean ± SD of the mean. ^∗^*P* < 0.05, ^∗∗^*P* < 0.01, ^∗∗∗^*P* < 0.001.

The administration of P5 hUC-MSCs to rats with injured livers is more effective than P10 hUC-MSCs. Importantly, primary MSCs lose ligands or receptors that respond to migratory signals during *in vitro* expansion [16]. Therefore, we assessed the homing efficacy of P5 and P10 hUC-MSCs with regard to injured livers. To investigate the homing efficacy, CM-Dil-labeled P5 and P10 hUC-MSCs (1 × 10^6^ cells) were engrafted via tail vein 24 h after the injection with D-GalN/LPS. As shown in Figure 6(a), the engrafted cells mainly distributed around the central vein and in the sinusoids. After P5 hUC-MSC administration, the number of cells with fluorescence (120 ± 14) was higher than for P10 hUC-MSC administration (77 ± 12) in the liver (Figure 6(b)). These data suggested that P5 hUC-MSCs migrated more efficiently to the injured livers.

### 3.7. c-Met Expression in hUC-MSCs

**Figure 7:**
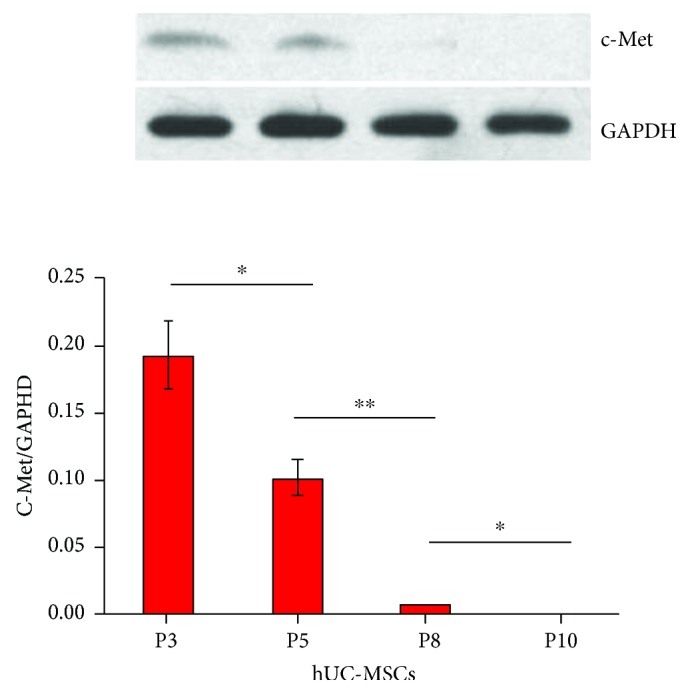
The protein levels of c-Met were analyzed by Western blotting in P3, P5, P8, and P10 hUC-MSCs. Data are presented as mean ± SD. ^∗^*P* < 0.05, ^∗∗^*P* < 0.01.

Our primary experiment found that levels of HGF in the liver significantly increased and peaked at 24 h and 48 h, respectively, post-D-GalN/LPS injection. In addition, overexpressed c-Met in BM-MSCs improved the homing efficacy to injured livers and improved liver function. Furthermore, freshly isolated MSCs lose ligands or receptors that respond to migratory signals during expansion [13, 16]. The c-Met protein levels in hUC-MSCs of P3, P5, P8, and P10 generations were analyzed via Western blotting. As the number of cell passages increased, the c-Met expression level of hUC-MSCs gradually decreased. There was a significant difference in the expression of c-Met between P5 and P8 hUC-MSCs, while c-Met expression in P10 hUC-MSCs could barely be measured (Figure 7). These results seem to be indicative of the better homing efficacy of the P5 hUC-MSCs, and the enhanced homing efficacy of the P5 hUC-MSC transplantation noticeably improves liver function.

## 4. Discussion

With the capacity of self-renewal and multipotency, MSCs have been increasingly used in tissue engineering and regenerative medicine [17]. BM-MSCs have been thoroughly characterized [18, 19]. However, their acquisition can be harmful, and their differentiation capability decreases with age [20].

In a previous study, UC-MSCs were successfully isolated and cultivated [21]. Due to the ease of accessibility, low immunogenicity, and higher differentiation capacity, hUC-MSCs have been investigated as an ideal clinical source of MSCs for cell therapy [21, 22]. The present study was conducted to validate the characteristics of hUC-MSCs. The cells formed a morphologically homogeneous population of fibroblast-like cells, when cultured in vitro. Flow cytometry indicated that the cells were positive for CD105, CD90, and CD44, but negative for CD34, CD31, and CD45. The results suggest that these cells have a mesenchymal origin, which is consistent with previous reports [23, 24]. During P5 hUC-MSC transplantation, the mortality of rats with ALF was effectively reduced by 62.5%, compared with the control. Furthermore, the serum levels of AST, ALT, and TBil were significantly decreased after treatment with P5 hUC-MSCs, which was superior to P10 hUC-MSC transplantation. Liver histopathology and pathological scores were assessed to evaluate the effectiveness of hUC-MSC transplantation. Histological evaluation of the liver tissue after cell administration provided initial insights into the cellular targets of this therapy. The results suggest that hUC-MSCs may reconstitute liver tissue.

ALF is typically associated with progressive, massive hepatocellular necrosis [25]. Although UC-MSCs can differentiate into hepatocytes *in vivo*, the proportion of hepatocytes derived from hUC-MSCs was <1% of the total hepatocytes. The data suggested that the differentiated hepatocytes may not compensate for the recovery of liver function [26]. Stimulation of endogenous regeneration represents another potential mechanism of an MSC-based therapeutic effect, previously described in models of myocardial infarction [27]. Our results demonstrated that the delivery of hUC-MSCs can increase the number of proliferating hepatocytes. The P5 hUC-MSC therapy increased PCNA nearly twofold compared to the control group, while the P10 hUC-MSCs had a more moderate increase. Furthermore, inhibition of cell death is one therapeutic basis of MSC therapy that has been observed in models of myocardial infarction and stroke [28, 29]. In this study, we also provided evidence that the delivery of hUC-MSCs has the potential to reduce hepatocyte apoptosis. This correlated with observations in previous studies involving CCL4-induced liver injuries, where hUC-MSC therapy inhibited hepatocellular apoptosis and stimulated liver regeneration *in vivo* [30]. It is generally known that MSCs secrete trophic factors and cytokines, acting in a paracrine role as a mediator, involving antiapoptotic factors (HGF and insulin-like growth factor-1 (IGF-1)), angiogenetic factors (vascular endothelial growth factor (VEGF)), and mitogenetic factors (epidermal growth factor (EGF) and HGF) [31]. Recently, it has been recognized that MSCs release numerous extracellular vesicles (EVs) that participate in tissue regeneration via transferring information to damaged cells or tissue and exert biological activity similar to the MSCs [31]. Tan et al. showed that MSC-EVs increased hepatocyte regeneration by upregulating proteins associated with proliferation such as PCNA and cyclin D1 and the antiapoptosis gene Bcl-xL [32]. Moreover, Tamura et al. evaluated that MSC-EVs inhibited the secretion of proinflammatory cytokines and also increased the release of anti-inflammatory cytokines and the level of regulatory T cells [33]. In addition, MSC-EVs modulated the inflammatory response and activated antiapoptotic pathways via specific RNAs [34].

Most reports describe cell transplantation as the primary mode of MSC therapy, but the therapeutic effect of MSCs depends on the homing efficacy to the injured sites. HGF is the most effective mitogen in hepatocyte regeneration during tissue injury, and its biological effects rely on tyrosine kinase receptors and on c-Met. The HGF-c-Met axis played a crucial role in the homing of MSCs during liver injury [11].

Our primary experiment demonstrated that the highest concentration of HGF was observed in the liver at 24 h post-DGalN/LPS coinjection. Meanwhile, overexpressed c-Met in BM-MSCs improved the homing efficacy to the injured liver [12].

In addition, during expansion, primary MSCs lose ligands or receptors that respond to migratory signals [13, 16]. Our results suggested that P5 hUC-MSCs migrate more efficiently to the injured liver, compared to P10 hUC-MSCs. In addition, our results showed that the c-Met expression was dependent on the cell passage number. Higher levels of c-Met may correlate with the better homing efficacy of P5 hUC-MSCs when compared to P10 hUC-MSCs. Despite higher c-Met expression in P3 hUC-MSCs, the limited number of P3 hUC-MSCs collected cannot achieve the required number for delivery. Even if the required number of MSCs for treatment could be achieved, the economic costs also need to be taken into account. As a result, P5 hUC-MSCs may be the ideal alternative for cell therapy. Furthermore, G. Wang and P. Wang demonstrated that hypoxia plays an important role in the increase of c-Met expression and the invasiveness of human prostatic carcinomas. Invasion of DU 145 cells (human prostate cells) in hypoxic conditions correlates with the expression level of c-Met [35]. Whether hypoxia promotes the c-Met expression in P5 hUC-MSCs needs to be further verified.

Migration within the injured tissues is influenced by multiple factors. Administration of the MSCs via the portal vein or hepatic artery shows homing efficacy less than 5% and 20–30%, respectively [36, 37]. Moreover, hypoxia induces the expression of leptin, which augments the recruitment of MSCs to the liver [38]. Meanwhile, more than 60 different microRNAs in MSCs have recently been described, and some of them are involved in migration, including let7, microRNA-10b, microRNA-27b, microRNA-335, and microRNA-886-3b [39]. Moreover, the loss of SH3, ICAM, and integrin-1 has been reported in the production of extracellular molecules accompanied with the culture of primary human MSCs [40].

## 5. Conclusions

Recent studies have detailed the potential benefit of hUC-MSC infusion for the treatment of ALF. The administration shows that systemic hUC-MSC therapy has profound inhibitory effects on hepatocellular apoptosis and stimulates liver regeneration for compensated liver function and also improves survival rates in rats with D-GalN/LPS-induced ALF. Furthermore, our results suggested that P5 hUC-MSCs migrate more efficiently to the injured liver, compared with P10 hUC-MSCs. The primary mechanism seems to be the regulation of homing molecules on MSCs during incubation *in vitro*, such as chemokine receptors or adhesion molecules, which has also been shown to affect the homing of HSCs [41, 42]. However, we can only presume that the rapid decrease in MSC homing efficiency *in vivo* is dependent on the level of c-Met.

Thus, hUC-MSCs may be a potential alternative source for cell therapy of ALF and may additionally mitigate the shortage of hepatocytes.

## Data Availability

The data used to support the findings of this study are included within the article.

## Abbreviations

MSCs: Mesenchymal stem cells
hUC-MSCs: Human umbilical cord mesenchymal stem cells
ALF: Acute liver failure
D-GalN: D-galactosamine
HGF: Hepatocyte growth factor
C-Met: Cellular mesenchymal to epithelial transition factor
AST: Aspartate aminotransferase
TBil: Total bilirubin
ALT: Alanine aminotransferase
EVs: Extracellular vesicles
PCNA: Proliferating cell nuclear antigen
TUNEL: Terminal deoxynucleotidyl transferase-mediated nick-end labeling
IGF-1: Insulin-like growth factor-1
VEGF: Vascular endothelial growth factor
EGF: Epidermal growth factor.
